# Different Nodules Identified during Liver Explant Gross Examination: Relevance and Need for Sectioning—Experience from India

**DOI:** 10.1155/2016/4390434

**Published:** 2016-06-22

**Authors:** Nalini Bansal, Vivek Vij, Mukul Rastogi

**Affiliations:** ^1^SRL Ltd., Fortis Escorts Heart Institute, New Delhi, India; ^2^Fortis Hospital, Gurgaon, India

## Abstract

*Objective*. The goal of this study was to determine the etiopathological association of various hepatic nodules identified during gross examination of liver explants specimen and the grossing aspects of these abnormal nodules especially those smaller than 1 cm in diameter. Our aim was to analyze whether there is any association of macroregenerative and dysplastic nodule with hepatocellular carcinoma.* Materials and Methods*. Fifty consecutive liver explants specimens were analyzed for the presence of any abnormal nodule (abnormal nodule defined as any nodule different in color, texture, and appearance from adjacent liver tissue).* Results*. Of the total 40 abnormal nodules identified in 50 liver explant specimens, there were 12 (30%) HCC [including 5 small HCC (41% of total HCC) and 1 steatohepatitic HCC (8% of total HCC)], 11 (27%) MRNs, 8 (20%) dysplastic nodules, and 9 (22%) necrotic nodules. Most cases (72%) of MRN are seen in hepatitis C virus related cirrhosis with only 2 cases having associated HCC. Most cases of HCC were seen in cases of HBV associated cirrhosis (60%). The association of MRN was not found to be significantly associated with HCC with a *p* value of 1.0. Dysplastic nodules were found to be significantly associated with HCC with a *p* value of 0.02.* Conclusion*. In hepatic carcinogenesis, the role of MRN does not appear to be significant. However, the presence of dysplastic nodules is significantly associated with HCC. The study identified another variant of cirrhotic nodules herein called necrotic nodules that are mostly tan greenish in color and <0.5 cm in diameter. No dysplastic changes were identified in any of these nodules disqualifying the need of sectioning in such nodules.

## 1. Introduction

MRN and dysplastic nodules are increasingly being recognized as important aspects of hepatocellular carcinoma. The results of recent investigations from Japan, America, and Europe have suggested that the old hypothesis of dysplasia-carcinoma sequence in liver needs to be qualified. The identification of this precancerous lesion may aid in prevention and timely management of such patients. Future studies must show whether and if so which immunohistochemical or molecular-genetically detectable changes can be utilized as risk markers in the diagnostic workup of these lesions. With this background, this study was undertaken to identify the distribution of various nodules in explant liver and to analyze their role in hepatic carcinogenesis.

## 2. Material and Methods

From May 2015 to September 2015, 50 liver explant operations were performed at our institution. These explant specimens were analyzed for the presence of any abnormal nodules. Abnormal nodules were defined as nodule of any size, which is different in color, texture, and appearance from surrounding liver parenchyma. These abnormal nodules were histologically classified according to International Working Party classification 1995 as MRN, dysplastic nodule, HCC its variants, and small HCC. The statistical analysis was performed using Fisher's Exact two-tailed tests.

The three major hepatic veins of all explant specimens were opened after inserting a probe and they were thereafter serially sliced at 0.5–1 cm interval; any abnormal nodule identified was sectioned other than the sections taken routinely as three from the right lobe, two from the left lobe, one from the caudate lobe, one from the porta hepatis, and one from the gall bladder. All sections were stained routinely with Haematoxylin and Eosin stain. Special stains including Masson's trichrome, PAS, PAS with diastase, Orcein, Pearl, and reticulin stain were performed as and when required.

Histologically, these nodules were classified according to the International Working Party Classification 1995 as macroregenerative nodules, dysplastic nodules, hepatocellular carcinoma, and small hepatocellular carcinoma.

## 3. Results

A total of 40 nodules were identified in 50 explant liver specimens examined. The ages of patients ranged from 9 to 68 years with a male : female ratio of 5 : 1. Of the total 40 abnormal nodules identified in 50 liver explant specimens, there were 12 (33%) hepatocellular carcinomas [including 5 small HCC (41% of total HCC) and 1 steatohepatitic HCC (8% of total HCC)], 11 (27%) macroregenerative nodules, 8 (20%) dysplastic nodules, and 9 (22%) necrotic nodules. Of the HCC group, there were 5 small HCC measuring <2 cm in size and one was steatohepatitic HCC (SH-HCC).

The commonest etiology of cirrhosis in explant liver specimens in this part of the world was hepatitis C virus (HCV) seen in 40% of all cases followed by hepatitis B virus (18%) and alcohol (14%) (Table 1).

### 3.1. Hepatocellular Carcinoma

HCC were seen in 5/9 cases of hepatitis B virus associated cirrhosis compared to 5/20 cases of hepatitis C virus associated cirrhosis. Only 1 case of HCC was seen in NASH associated cirrhosis and one was cryptogenic. The association of HCC with HBV was significant with a *p* value of 0.027. Alpha-fetoprotein levels were elevated in 11/12 cases of HCC. Of all HCC cases, there was 1 case of steatohepatitic HCC seen in case of HCV cirrhosis. This patient was a 49-year-old male known to be hypertensive and diabetic for the last 12 years and had been on regular medication. His lipid profile showed serum cholesterol of 230 mg/dL (<200) and triglycerides of 150 mg/dL (35–150), HDL cholesterol of 44 mg/dL (35–85), and LDL cholesterol of 115 mg/dL (<150). The patient had metabolic syndrome with hepatitis C virus associated cirrhosis. Grossly, the nodule measured 2.5 × 2 cm with a yellowish cut surface. Microscopy showed steatohepatitic HCC with prominent ballooning and steatosis and adjacent liver showed cirrhosis with mild steatosis.

#### 3.1.1. Small Hepatocellular Carcinoma

**Figure 1:**
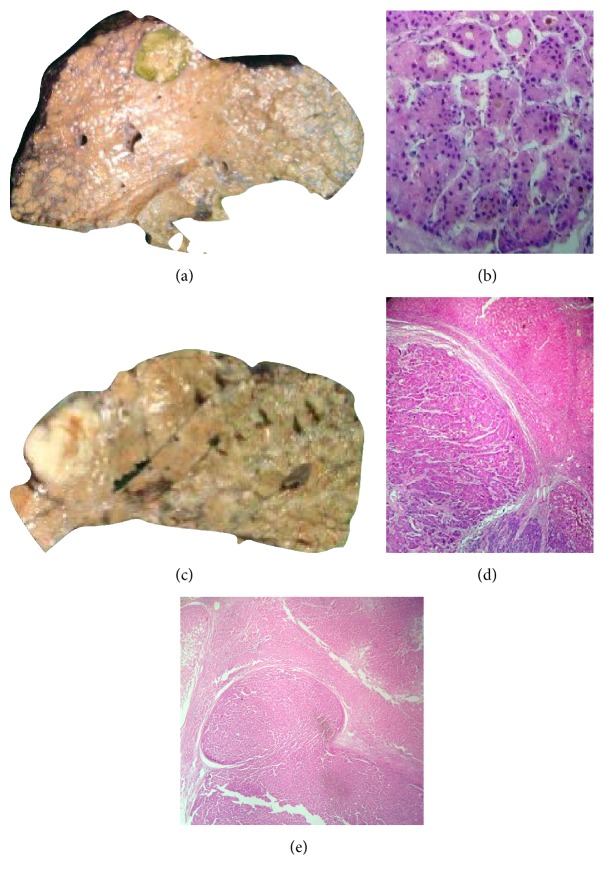
(a) Small HCC in liver explant. (b) Photomicrograph showing neoplastic hepatocytes in pseudoacinar pattern (H&E ×40). (c) Small HCC in liver explant. (d) Photomicrograph showing thick trabeculae of neoplastic hepatocytes (H&E ×20). (e) Mushrooming phenomenon of small HCC.

Grossly, all cases presented with nodule different in color from adjacent liver tissue measuring 0.8–2 cm in diameter. Diagnosis of small HCC was based on absence of portal tracts, loss of reticulin fibres, and presence of thick trabeculae and pseudoacinar pattern of atypical hepatocytes (Figures 1(a) and 1(b)). AFP was found to be minimally to mildly elevated in all 5 cases of small HCC (9–129 ng/mL) but in none of the cases was it above 500 ng/mL (Table 2). Preoperative triple phase Contrast Enhanced CT has a high diagnostic accuracy (80%) of diagnosing small hepatocellular carcinoma. 80% of cases of small HCC were seen in right lobe and measured 1-2 cm in size. All these cases were diagnosed preoperatively by triple phase CECT. One case in left lobe measuring 0.9 × 0.8 cm could not be diagnosed preoperatively. In one case of small HCC, there were associated dysplastic nodules of both low- and high-grade type. This patient had previous partial hepatectomy for HCC and presented with multiple liver nodules after a year of follow-up in residual liver tissue.

*Another Interesting Finding Was Noted in Few Cases of Small HCC*

*Mushroom Effect.* There was bullous protrusion at one end of the neoplastic nodule probably representing the origin of these small HCC resembling a hand mirror herein called mushrooming phenomenon.

### 3.2. Macroregenerative Nodules

**Figure 2:**
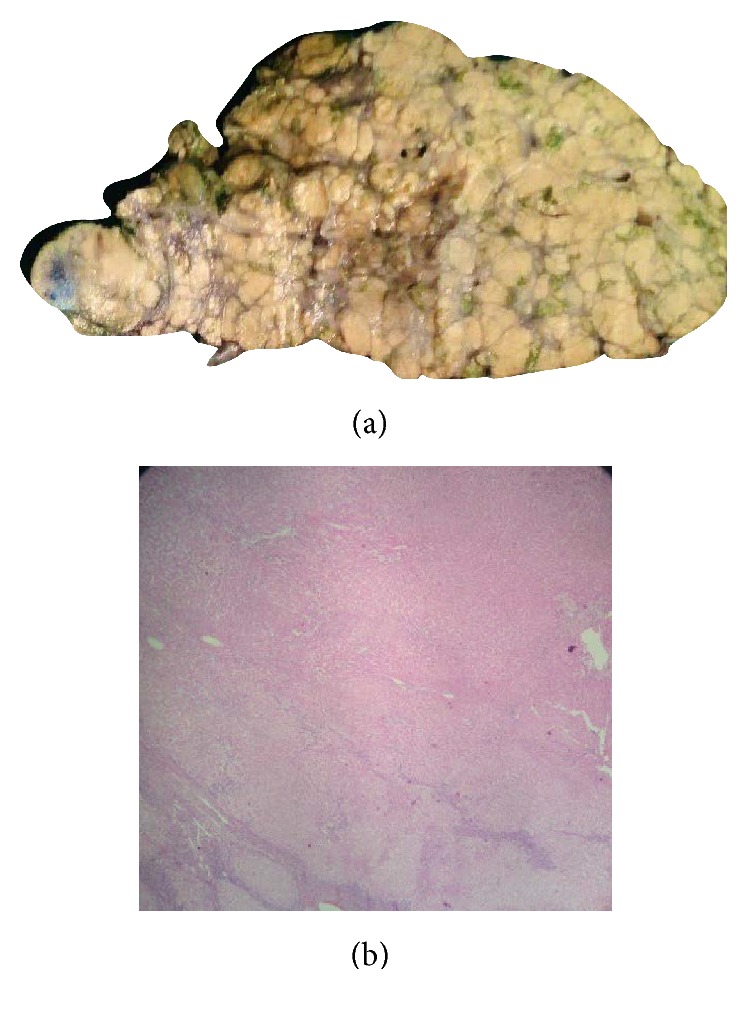
(a) Macroregenerative nodules in explants liver. (b) Photomicrograph showing multiacinar MRN (H&E ×20).

MRNs were observed in 27% of cases of total explant specimens (Figures 2(a) and 2(b)). Most of these nodules were similar in color to surrounding liver with size >1 cm. Microscopy in all showed multiacinar cirrhotic nodules with several portal tracts. In only 2 cases, MRN was seen along with HCC. One of these cases also showed presence of dysplastic nodule along with HCC. No significant association was seen between macroregenerative nodule and HCC and the *p* value was not significant.

### 3.3. Dysplastic Nodules

**Figure 3:**
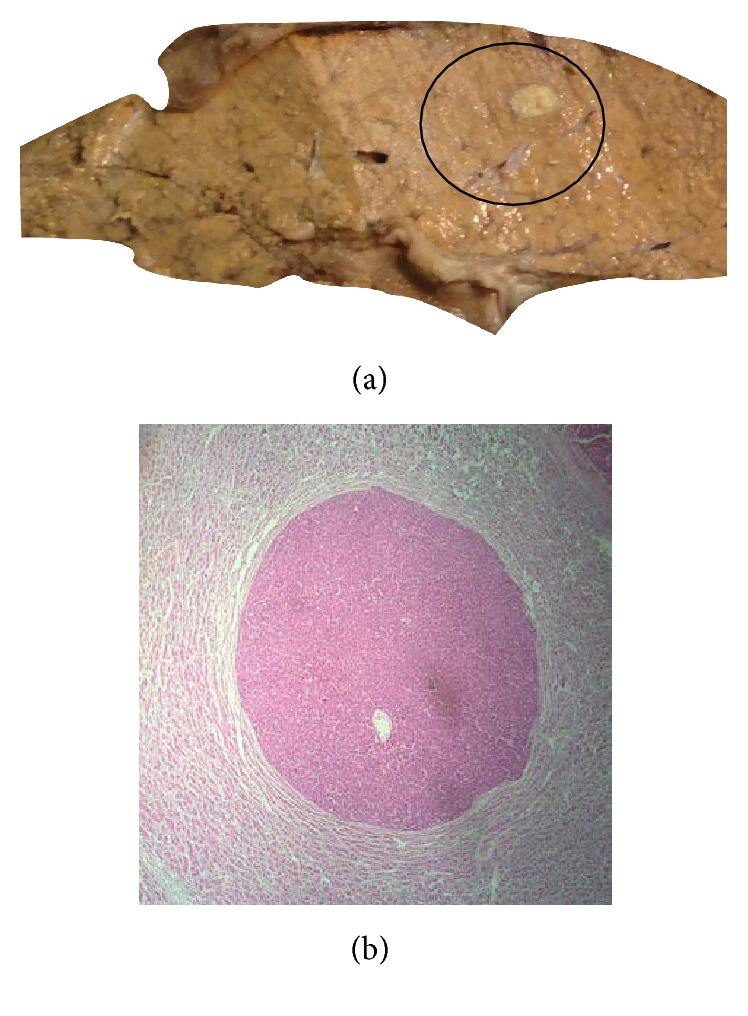
(a) Dysplastic nodule in explant liver. (b) Photomicrograph showing dysplastic nodule with small cell change (H&E ×10).

There was a significant association of the presence of dysplastic nodules with HCC with a *p* value of 0.02. The dysplastic nodules grossly were of different color or texture from adjacent liver. Microscopy showed hepatocytes with small or large cell change and scanty portal tracts. Hepatocytes are usually single cell plate thick. Of these 8 dysplastic nodules, there were 6 high-grade dysplastic nodules and 2 low-grade dysplastic nodules showing mild atypia. Five of these nodules (3 of high grade and 2 of low grade) were seen in a single explant with small HCC (Figures 3(a) and 3(b)). Small cell change was seen in 5 of these nodules.

### 3.4. Necrotic Nodules

**Figure 4:**
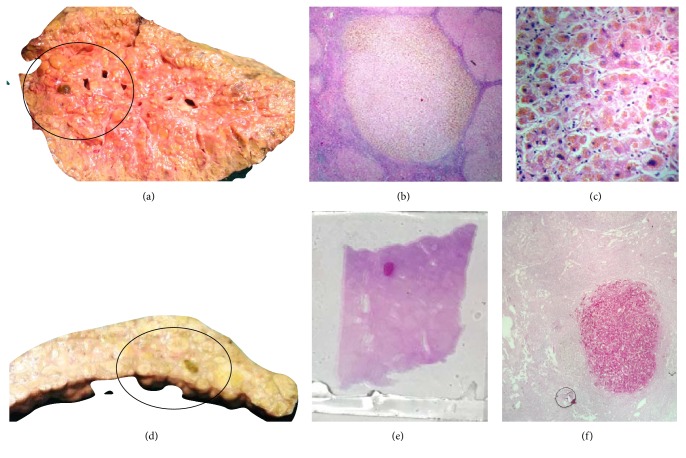
(a) Necrotic nodule in explant liver (b and c). Photomicrograph showing all degenerated hepatocytes in low and high power (H&E ×10 and 40). (d) Necrotic nodule in explant liver (e and f). Photomicrograph showing PAS positive staining of necrotic nodule in section and low power.

There is no literature so far on the existence of these nodules. All of these were discolored, greenish yellow, and small (<0.5 cm) in diameter (Figures 4(a), 4(b), 4(c), and 4(d)). Most (87%) of these nodules were identified in HCV related cirrhosis. Microscopy of these nodules showed mainly degenerated hepatocytes with prominence of lipofuscin pigment that is PAS positive. No evidence of dysplasia was identified in any of these nodules.

## 4. Discussion

Distribution of various nodules found on liver explant gross examination has long been a matter of debate especially for evaluating their role in hepatic carcinogenesis. The whole idea is to diagnose these hepatic carcinomas in their precursor stages and treat them early before full-blown malignancy develops. There has been scant literature on etiology and pathogenesis from South Asia and developing countries mainly because of the lack of registries of liver and other diseases in our population. We found that HCV is the most common etiology of liver cirrhosis in South Asia seen in 40% of cases of explant liver followed by hepatitis B virus and alcohol. A Japanese study on 345 patients states that hepatitis B virus infection appears to be a frequent cause of cirrhosis of the liver and hepatocellular carcinoma in Asia and Africa [1].

Another study from Malaysia on 460 patients also states that the major causes of cirrhosis were chronic hepatitis B (46.1%) followed by chronic hepatitis C (18.5% of cases) [2]. These are in contrast to our group of patients where hepatitis C virus is the major etiological association with liver cirrhosis.

Of these cases of liver cirrhosis, the major etiological association of hepatocellular carcinoma was with hepatitis B virus and the relation was found to be significant with a *p* value of 0.027.

Chronic HBV infection has been implicated as a dominant risk factor for HCC in most areas of Asia and sub-Saharan Africa with the exception of Japan, where the major risk factor for HCC is chronic HCV infection [3].

Taking a cut-off of 500 ng/mL, the diagnostic accuracy of AFP was very low for small HCC with all cases having AFP < 500 ng/mL. However, in all cases, the value of AFP was higher than normal (>9 ng/mL) ranging from 9 to 129 ng/mL compared to cirrhotics without HCC where AFP was in normal range. In a resource constrained country where people cannot afford to get many tests done, even minor elevation of AFP should be considered to be significant in known cirrhotics and they should undergo radiological evaluation. Other studies have shown that diagnostic accuracy of AFP in small HCC was substantially limited taking a cut-off of >500 ng/mL to be of diagnostic utility. They have emphasized the role of newer markers in early diagnosis of HCC like AFP-L3, prothrombin induced by vitamin K absence-II (PIVKA-II), which is an abnormal prothrombin protein that is present at higher levels in the serum of HCC patients [4].

Relationships of MRN and HCC have been studied extensively in the last decade with major studies coming from the Japanese and American group. The Japanese group initially proposed that MRN could be involved in the morphogenesis of HCC from autopsy studies in patients of chronic liver diseases initially being performed on 345 patients [5].

Also, other studies from Japan [6] on 141 liver explants identified similar large regenerative nodules. They identified 94 large regenerative nodules in 53 cirrhotics. Further studies by Japanese groups [7] on 209 cirrhotics identified ordinary and atypical adenomatous hyperplasia (AAH) in these cirrhotics and concluded that AAH may be an important preneoplastic lesion in cirrhotic livers associated with non-A non-B hepatitis virus (probably hepatitis C virus). They also conducted a follow-up study for these atypical adenomatous nodules, which were resected surgically. They classified these adenomatous hyperplasia nodules as ordinary adenomatous hyperplasia (OAH) lacking hepatocellular atypia, atypical adenomatous hyperplasia with structural and cellular atypia insufficient for carcinoma (AAH), and atypical AH with focal malignancy containing areas of HCC (FM). On follow-up of these patients (follow-up period range: 12–77 months; mean: 31.4 months), HCC was seen in all 3 patients whose resected nodules were classified as FM, in 4 (36%) of 11 with AAH resected nodules, and in none of 10 with OAH resected nodules. The incidence of HCC in the patients with focal malignancy (FM) or AAH nodules was found to be significantly higher than that in those with OAH nodules, thus emphasizing their preneoplastic nature [8].

American groups, mainly the work by Theise et al., in their initial work on 44 explant specimens found 48 MRNs in 11 explants. They concluded that the presence of MRN is more common in non-Japanese cirrhotic patients occurring in several different types of liver diseases and representing precancerous lesions [9].

In the subsequent years, their further study on 155 explant specimens (inclusive of previous study on 44 explants), they again concluded that the presence of either type of MRN (type I: without dysplasia; type II: with dysplasia) was associated with an increased incidence of HCC (all MRNs, *p* < 0.00019; type I MRNs, *p* < 0.067; type II MRNs, *p* < 0.012) compared with cirrhotic livers without MRNs [10].

Another study by Ferrell et al. analyzed 110 liver explant specimens with 28 MRNs with HCC seen in 3 of them. They also proposed a possible role of MRN in HCC [11].

A group from France examined 41 consecutive cirrhotic liver explants from French patients. Thirty-five adenomatous hyperplasias were identified in 10 livers (prevalence: 24%); seven of 10 were HCV positive. Their data also suggest that hepatocarcinogenesis is a multistep process and AAH should be considered as a premalignant lesion whereas OAH had proliferative ability [12].

Studies from African countries have concluded that MRN could not be considered a risk factor for HCC [13].

Our study being the first from the Indian subcontinent also highlighted similar findings with macroregenerative nodules not significantly associated with HCC whereas dysplastic nodules have significant association with HCC with a *p* value of 0.02 compared to cirrhotic liver without dysplastic nodules, thus emphasizing that dysplastic nodules are involved in tumor carcinogenesis of HCC.

SH-HCC is a recently described variant of HCC with only 3 series and 1 case report reported in the literature. Salomao et al. have proposed histological criteria for diagnosis of SH-HCC. The criteria state that 3 of 5 features are required in >50% of the tumor mass to be diagnosed as SH-HCC. The five features included were steatosis, ballooning degeneration, Mallory Denk Bodies, inflammation, and pericellular fibrosis. Most of these variants are seen to develop in the background of metabolic syndrome or HCV cirrhosis. In our case, the patient had both as reported by other authors [14–17].

*Necrotic Nodules.* These nodules were identified in 9 of 50 liver explant specimens. These nodules were macroscopically seen as greenish yellow nodules mostly <0.5 cm and microscopically composed of lipofuscin pigment containing hepatocytes. None of these nodules showed features of dysplasia or malignancy. Most of these nodules (87%) were seen in HCV related cirrhosis. The literature is quiet on the presence of these nodules in explant liver.

*Mushroom Effect of Small HCC*. Small HCC have been identified to show mushrooming phenomenon seen erupting out from a small bud of neoplastic cells at one of the foci. Similar morphological observations have not been previously reported in the literature.

Recently, several genetic studies have been undertaken to determine whether there are any molecular markers for precarcinogenesis in cirrhotic nodules. Studies by Nault et al. analyzed a series of 268 liver samples for telomerase reverse-transcriptase promoter (TERT) mutations. They found that TERT mutations were highly related to the stepwise hepatocarcinogenesis. They concluded that TERT promoter mutation is the most frequent and also the earliest genetic alteration in early HCC [17, 18].

## 5. Conclusion

Our study from the Indian subcontinent also supports the hypothesis that dysplasia precedes carcinoma and may represent precursor lesion of HCC. MRNs are not significantly associated with HCC. Necrotic nodules identified on explant liver need not be sectioned in explant specimen though the findings need to be substantiated on a larger cohort of patients.

## Figures and Tables

**Table 1:** Etiopathological association of explant liver and HCC.

Etiology	Number	Percentage	HCC
HCV	20	40%	5
HBV	9	18%	5
Alcohol	7	14%	—
Cryptogenic	4	8%	1
PBC	2	4%	—
Mixed HBV & HCV	3	6%	—
Wilson	2	4%	—
NASH	1	2%	1
PFIC	1	2%	—
MLD	1	2%	—
Mixed HBV & HCV	3	6%	—

**Table 2:** Etiopathological association of small HCC with AFP level and triple phase CECT.

S. number	Age	Etiology	AFP (0.2–9 ng/mL)	Histology	Radiology	Concordance
1	46 M	HBV	9.6	2 × 1.5 × 1.5 cm RL	Seg. VIII, 1.8 × 1.5 cm	C
2	68 M	Cryptogenic	13.6	1.6 × 1.5 cm RL	Seg. IV A, 15 × 14 mm	C
3	49 M	HCV	12.8	0.9 × 0.8 cm LL	No lesion	D
4	55 M	HCV	129	2 × 1.8 RL	Seg. VIII, 2 × 2 mm	C
5	47 F	HCV	9.7	1.5 × 1.4 cm	Seg. VIII,16 × 13 mm	C
